# Surface Modification of Super Arborized Silica for Flexible and Wearable Ultrafast‐Response Strain Sensors with Low Hysteresis

**DOI:** 10.1002/advs.202301713

**Published:** 2023-06-28

**Authors:** Shaowei Han, Huanhuan Tan, Jia Wei, Hang Yuan, Songwei Li, Peipei Yang, Haoyang Mi, Chuntai Liu, Changyu Shen

**Affiliations:** ^1^ National Engineering Research Center for Advanced Polymer Processing Technology Key Laboratory of Materials Processing and Mold (Ministry of Education) Zhengzhou University Zhengzhou 450002 China; ^2^ Yunnan Tobacco Quality Inspection and Supervision Station Kunming 650106 China

**Keywords:** low hysteresis, nanocomposite conductive hydrogels, strain sensors, super arborized silica

## Abstract

Conductive hydrogels exhibit high potential in the fields of wearable sensors, healthcare monitoring, and e‐skins. However, it remains a huge challenge to integrate high elasticity, low hysteresis, and excellent stretch‐ability in physical crosslinking hydrogels. This study reports the synthesis of polyacrylamide (PAM)‐3‐(trimethoxysilyl) propyl methacrylate‐grafted super arborized silica nanoparticle (TSASN)‐lithium chloride (LiCl) hydrogel sensors with high elasticity, low hysteresis, and excellent electrical conductivity. The introduction of TSASN enhances the mechanical strength and reversible resilience of the PAM‐TSASN‐LiCl hydrogels by chain entanglement and interfacial chemical bonding, and provides stress‐transfer centers for external‐force diffusion. These hydrogels show outstanding mechanical strength (a tensile stress of 80–120 kPa, elongation at break of 900‐1400%, and dissipated energy of 0.8–9.6 kJ m^−3^), and can withstand multiple mechanical cycles. LiCl addition enables the PAM‐TSASN‐LiCl hydrogels to exhibit excellent electrical properties with an outstanding sensing performance (gauge factor = 4.5), with rapid response (210 ms) within a wide strain‐sensing range (1–800%). These PAM‐TSASN‐LiCl hydrogel sensors can detect various human‐body movements for prolonged durations of time, and generate stable and reliable output signals. The hydrogels fabricated with high stretch‐ability, low hysteresis, and reversible resilience, can be used as flexible wearable sensors.

## Graphical abstract

A synthesized surface‐modified super arborized silica nanoparticles (TSASNs)‐polyacrylamide (PAM)‐lithium chloride (LiCl) ultrafast response strain sensor with high fracture toughness and low hysteresis is prepared. The hydrogel sensor can rapidly and stably detect different types of human movement. The work contributes immensely to the field of flexible wearable sensors.

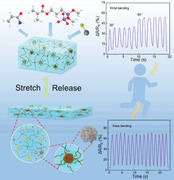

## 1 Introduction

Flexible electronic devices have garnered immense attention due to their high potential in healthcare monitoring, for manufacturing e‐skins, and as a human–machine interface.^[^ ^1^ ^]^ They sense external stimuli, such as pressure,^[^ ^2^ ^]^ strain,^[^ ^3^ ^]^ and temperature,^[^ ^4^ ^]^ and convert them into electrical signals. Hydrogels, hydrophilic 3D network‐structure gels with good biocompatibility and super elasticity,^[^ ^5^ ^]^ are promising candidates for wearable electronics.^[^ ^6^ ^]^ However, conventional hydrogels frequently rupture during stretching, due to their weak mechanical properties; this limits their practical application. In recent years, several physical and chemical methods have been presented that improve the mechanical strength of hydrogels. However, high‐toughness hydrogels fabricated by introducing double networks,^[^ ^7^ ^]^ inorganic composite materials,^[^ ^8^ ^]^ and topological crosslinking^[^ ^9^ ^]^ exhibit high hysteresis. The trade‐off between fracture toughness and hysteresis makes it difficult to produce conductive hydrogels with excellent comprehensive properties.

Several strategies have been used to improve the toughness and hysteresis of hydrogels, such as polyprotein crosslinking, peptide crosslinking, and chain entanglement. Lei et al. have reported tough and low‐hysteresis hydrogels, crosslinked by tandem repeating proteins, in which the randomly coiled polymer acts as a permeable network, and the protein crosslinking agent unfolds on experiencing excess force at the fracture zone, thereby preventing crack growth.^[^ ^10^ ^]^ Liu et al. have designed a tough and elastic hydrogel with reasonable peptide crosslinking; under stress, the protein helix is stretched into a long and thin chain, disrupting the intramolecular hydrogen bonding that stabilizes the helix structure, providing an alternative mechanism for energy dissipation.^[^ ^11^ ^]^ Kim et al. have designed a polymer with single‐network chain entanglement. Single‐network polymers are composed of long chains with a large number of chain tangles, which make the polymer resilient. On stretching a highly entangled polymer, the tension is transferred from one chain to another through the tangles, causing negligible polymer hysteresis.^[^ ^12^ ^]^ Notably, the aforementioned studies involve complex syntheses; therefore, it is vital to develop a facile method for the synthesis of high‐toughness and high‐resilience hydrogels, to facilitate the long‐term application.

Conducting nanomaterials, such as metals,^[^ ^13^ ^]^ graphene,^[^ ^14^ ^]^ MXene,^[^ ^15^ ^]^ and carbon nanotubes (CNTs),^[^ ^16^ ^]^ have been used as additives to fabricate hydrogels with reliable conductivity. Sun et al. have developed composite‐hydrogel (polyacrylamide (PAM) / multi‐walled carbon nanotubes (oxCNTs)) sensors. Although these composite hydrogels show a high tensile performance (up to 1000%), which is sufficient to monitor human‐body activity, the inherent hydrophobicity of the conductive material leads to the formation of aggregates in the internal network of the hydrogel, which adversely affects its conductive and mechanical properties.^[^ ^17^ ^]^ Moreover, opaque conductive materials generate opaque hydrogels, which cannot meet the diverse requirements of flexible electronic products.^[^ ^18^ ^]^ In large‐deformation processes, ion‐conductive hydrogels exhibit better reliability, mechanical properties, transparency, and sensitivity than hydrogels embedded with conducting nanomaterials.^[^ ^19^ ^]^ Jiang et al. have reported novel polyvinyl alcohol/polyacrylamide (PVA/PAM) double network (DN) hydrogels, and the addition of NaCl can induce chain‐entangled physical network formation. The PVA/PAM/NaCl hydrogels have both high conductivity and outstanding mechanical strength.^[^ ^20^ ^]^ On the basis of PVA/PAM DN hydrogels, Jiang et al. have designed a facile strategy to introduce 2‐acrylamido‐2‐methylpropane sulfonic acid (AMPS) and Glycerin (Gly). The presence of AMPS provides the composite hydrogels with excellent fatigue resistance and ionic conductivity. The Gly component endues hydrogels high adhesion and mechanical properties.^[^ ^21^ ^]^ Ding et al. have reported nano silica fiber‐reinforced hydrogels with robust covalent connections that exhibit excellent mechanical strength (a tensile stress of 0.3 MPa and fracture strain of 1400%). The introduction of NaCl improves the sensitivity of such hydrogel sensors significantly. However, they exhibit a large hysteresis (as indicated by tensile cyclic‐experiments), as the incorporation of nano silica fibers makes the hydrogel‐network flexible; this generates unstable signals, causing output‐signal inaccuracy.^[^ ^22^ ^]^ Moreover, unstable signals and inferior skin sensation are generated when non‐adhesive hydrogel sensors are attached to the human skin via tape or belt.^[^ ^23^ ^]^ Therefore, for high practicability, hydrogels should exhibit rapid and reversible resilience (low hysteresis), along with good mechanical strength, conductivity, and adhesion.

This paper proposes a strategy for the chain entanglement and interfacial chemical bonding of surface‐modified super arborized silica nanoparticles (TSASNs) with polyacrylamide (PAM), to prepare hydrogels with high fracture toughness and low hysteresis. TSASNs enhance the mechanical properties of PAM, possibly because the covalent‐crosslinking point acts as a stress‐transfer center. Moreover, the TSASN‐PAM contact area is large, and the entanglement of PAM with the TSASNs maintains the stability of the hydrogel network, which shows a low hysteresis. The introduction of LiCl to PAM‐TSASN_2_ hydrogels improves their mechanical properties and ionic conductivity, enabling the composite hydrogels to exhibit super sensitivity. Additionally, the ionic conductive hydrogels synthesized in this study are transparent and adhere to the human skin. PAM‐TSASN_2_‐LiCl_5_ hydrogels have been used to construct high‐performance sensors that can rapidly and stably detect different types of human movement. Therefore, the work contributes immensely to the field of flexible wearable sensors, and could facilitate their practical application.

## 2 Results and Discussion

### 2.1 Fabrication and Mechanism of PAM‐TSASN Hydrogels

**Figure 1:**
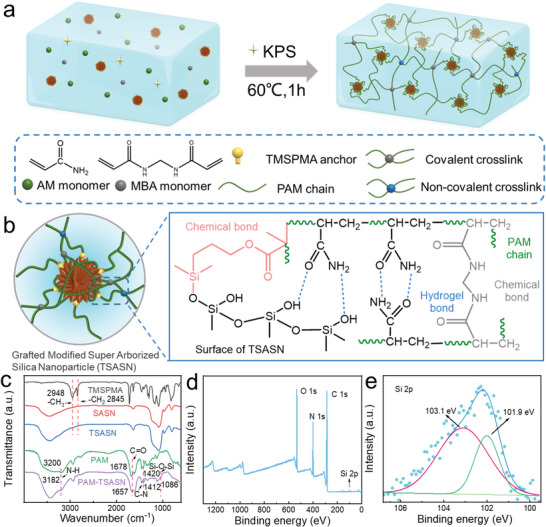
a) Schematic illustration of the fabrication process of PAM‐TSASN hydrogels. b) The hydrogel network and interactions. c) FTIR spectra of TMSPAM, SASN, and TSASN, and the PAM and PAM‐TSASN hydrogels. d) XPS spectrum of the PAM‐TSASN hydrogels. e) High‐resolution Si 2p XPS spectrum of the PAM‐TSASN hydrogels.

**Figure 2:**
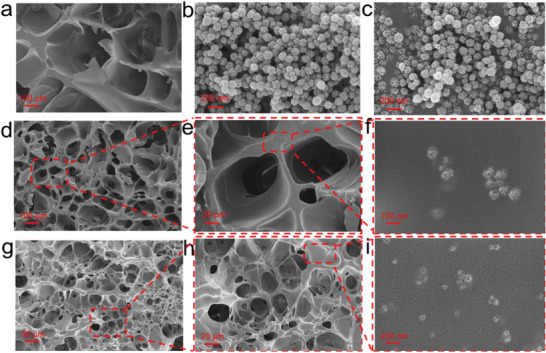
SEM images of freeze‐dried a) PAM hydrogels, b) SASN, c) TSASN, d–f) PAM‐TSASN_2_ hydrogels, and g–i) PAM‐TSASN_2_‐LiCl_5_ hydrogels.

As shown in **Figure** **1**a, hydrogels were prepared by a simple one‐step free radical polymerization. The reaction process of PAM‐TSASN and the TSASNs are schematically shown in Figure S1, Supporting Information. The SASNs and TSASNs showed a highly‐branched topology (**Figure** **2**b,c). The TSASN structure enabled PAM‐chain penetration, maximizing the contact area between the silica nanoparticles and polymer chains (Figures 1b and 2c); this facilitated the homogenous diffusion of external force at the stress‐transfer center.^[^ ^24^ ^]^ The PAM chains were covalently linked by N,N′‐methylene dimethyl (acrylamide) (MBA) and 3‐(trimethoxysilyl) propyl methacrylate (TMSPMA） (to silica nanoparticles, forming strong interfacial chemical bonds).^[^ ^22^ ^]^ Additionally, the silica nanoparticle‐PAM chain hydrogen bonding could be rapidly broken and recombined, enabling energy dissipation; this kept the hydrogel network intact during stretching (Figure 1b).^[^ ^25^ ^]^

In Figure 1c, the absorption peaks at 2948 and 2845 cm^−1^ could be ascribed to the stretching vibrations of the C—H bond of the methyl and methylene groups of TMSPMA, confirming TMSPMA grafting onto SASN.^[^ ^26^ ^]^ Moreover, the stretching‐vibration absorption bands at 3200, 1678, and 1420 cm^−1^ could be attributed to the N—H, C=O, and C—N bonds of PAM, respectively. All the characteristic bands of PAM shifted to lower frequencies in the PAM‐TSASN spectrum, this could be attributed to the formation of hydrogen bonds between PAM chains and TSASN;^[^ ^27^ ^]^ additionally, a new peak generated at 1086 cm^−1^ in the latter, which could be attributed to the Si—O—Si bond, confirming its formation in PAM‐TSASN.^[^ ^28^ ^]^

X‐ray photoelectron spectroscopy (XPS) was also used for the characterization of PAM‐TSASN. As shown in Figure 1d, XPS analysis confirmed the presence of silica nanoparticles in PAM‐TSASN; Si, O, C, and N were clearly detected in PAM‐TSASN. Moreover, in the high‐resolution Si 2p spectrum (Figure 1e), peaks due to SiO_4_ and the central silicon atom of the silane (R_3_SiO_1_) were detected at two different binding energies (103.1 and 101.9 eV, respectively),^[^ ^29^ ^]^ confirming TMSPMA grafting onto SASN. The Si 2p and O 1s peaks for TSASN were shifted to slightly lower binding energies in the PAM‐TSASN spectrum (Figure S3, Supporting Information), possibly due to robust molecular interactions caused by changes in the environment of the PAM‐TSASN hydrogel matrix and silica nanoparticles.^[^ ^22^ ^]^

SEM images (Figure 2a,d) indicate that, the PAM and PAM‐TSASN_2_ hydrogels exhibit a typical continuous microporous network, indicating an even dispersion of TSASN in the hydrogels. Unlike the PAM hydrogels with loose pores, the PAM‐TSASN_2_ hydrogels showed a dense pore structure. With the addition of TSASN, the average pore diameter reduced from 250 to 70 µm (Figure S2a,b, Supporting Information), possibly because TSASN acted as a physical crosslinking point, reducing the pore diameter and improving the mechanical properties of the system. Notably, the addition of TSASN did not destroy the porous network structure of the PAM‐TSASN_2_ hydrogels. Interestingly, the pore size of the PAM‐TSASN_2_‐LiCl_5_ hydrogels reduced further (to 10–30 µm) on LiCl addition (Figure 2g; Figure S2c, Supporting Information), and the mechanical properties of the system improved. Highly magnified SEM images indicate a uniform dispersion of TSASN on the surface of the hydrogels (Figure 2f,i).

### 2.2 Mechanical Properties of PAM‐TSASN Hydrogels

**Figure 3:**
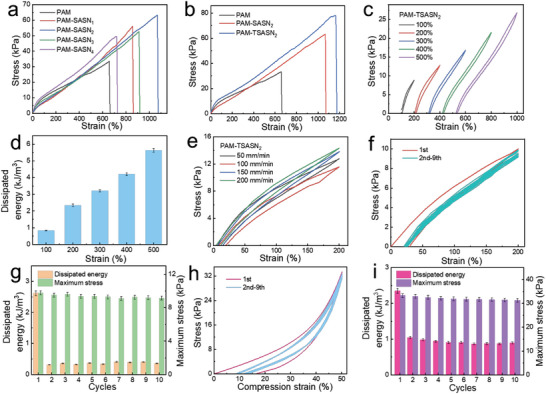
a) Tensile stress–strain curves of the PAM‐SASN*_x_* hydrogels. b) Tensile stress–strain curves of the PAM‐SASN_2_ and PAM‐TSASN_2_ hydrogels. c) Successive tensile cyclic‐curves of the PAM‐TSASN_2_ hydrogels at different strains and d) the corresponding dissipated energy (*n* = 3, *n* is the sample size for each group). e) Successive tensile cyclic‐curves of the PAM‐TSASN_2_ hydrogels at different stretching speeds. f) Cyclic tensile‐curves of the PAM‐TSASN_2_ hydrogels under a tension of 200% over 10 cycles, and g) the corresponding maximum stress and dissipated energy (*n* = 3, *n* is the sample size for each group). h) Cyclic compression curves of the PAM‐TSASN_2_ hydrogels under a compression of 50% over 10 cycles, and i) the corresponding maximum stress and dissipated energy (*n* = 3, *n* is the sample size for each group).

The mechanical properties of the PAM‐SASN*_x_* and PAM‐TSASN_2_ hydrogels were studied by uniaxial tensile and compressive tests. The tensile strain and stress of the PAM‐SASN*_x_* hydrogels were significantly greater than those of pure PAM hydrogels (700% and 35 kPa, respectively), and they increased on increasing the SASN content of the hydrogel, with a maximum value of 1070% and 63 kPa, respectively, as shown in **Figure** **3**a. This could be attributed to hydrogen bonding between the —OH in SASNs and —NH_2_ in PAM, and SASN providing physical crosslinking points in the PAM‐SASN*_x_* hydrogels. As the SASNs are very small nanoparticles, they possibly lubricated the PAM‐SASN*_x_* hydrogels and moved with the polymer chains during stretching, facilitating a wide range of flexible polymer‐chain movement. Increasing the SASN content of a PAM‐SASN*_x_* hydrogel softened its polymer chains, facilitating the formation of chain tangles with silica nanoparticles,^[^ ^30^ ^]^ thereby improving its elongation at break and fracture strength. However, hydrogels with SASN contents higher than 2 wt% showed low values of elongation at break and fracture strength, possibly due to a low physical crosslinking density. At extremely high concentrations, SASN accumulated in the PAM‐SASN*_x_* hydrogels, leading to a decrease in the number of silica nanoparticle‐polymer chain contact points. Subsequently, the compressive properties of the PAM‐SASN*_x_* hydrogels were investigated. On adding increasing amounts of the SASNs, the PAM‐SASN*_x_* hydrogels became increasingly flexible, and their compressive strength first decreased and then increased (Figure S4a, Supporting Information). This indicates that the SASNs, with small size and super arborized structure, imparted high fluidity to the PAM‐SASN*_x_* hydrogels during compression, facilitating compression‐stress dispersion; the mobile SASNs lubricated the PAM‐SASN*_x_* hydrogels, softening its polymer chains during the compression process.^[^ ^31^ ^]^ For SASN contents greater than 2 wt%, the PAM‐SASN*_x_* hydrogels aggregated, causing a reduction in the break stress, while the compressive stress increased, indicating a high crosslinking density and strong network rigidity.^[^ ^32^ ^]^ In order to improve the properties of the PAM‐SASN*_x_* hydrogels further, the SASNs were modified by grafting TMSPMA. This increased the break stress (80 kPa) and compression stress (200 kPa) of the PAM‐TSASN_2_ hydrogels (Figure 3b; Figure S4b, Supporting Information), possibly because the introduction of TMSPMA enabled strong interfacial chemical bonding between the PAM and SASNs. Figure 3c shows the stretching and relaxation curves of the PAM‐TSASN_2_ hydrogels at different strains; they were almost completely overlapping. On increasing the hydrogel strain from 100% to 500%, the dissipated energy improved slightly from 0.8 to 5.6 kJ m^−3^ (Figure 3d), indicating super elasticity and low hysteresis; this possibly due to a rapid reconstruction of the hydrogen‐bond network during tensile‐load release.^[^ ^33^ ^]^ The dissipated energy values of PAM‐SASN_2_ (Figure S4c,d, Supporting Information) and PAM‐TSASN_2_ (Figure 3c,d) were almost the same, indicating that the addition of TMSPMA improved the mechanical strength of the hydrogel, without affecting the dissipated energy. Notably, the dissipated energy changed negligibly on increasing the tensile speed (Figure 3e); this indicates that the PAM‐TSASN_2_ hydrogels are extremely stable. Thus, polymer‐chain motion and strong interfacial chemical bonding make the PAM‐TSASN_2_ hydrogels flexible, with high stretchability. Moreover, the hydrogen‐bond network in these hydrogels imparts excellent elasticity to the system, enabling its rapid recovery from distortion.

Anti‐fatigue behavior and reliability are important for the practical application of flexible sensors, because most tissues are subjected to repeated load‐unload cycles. The cycling performance of the PAM‐TSASN_2_ hydrogels was investigated under a constant strain of 200% for 10 consecutive load–unload tensile cycles (Figure 3f). A larger cyclic tensile curve was observed in the first load‐unload test, while the 2nd to 9th cyclic tensile curves remained stable, and almost overlapped. As shown in Figure 3g, during testing, the dissipation energy of the PAM‐TSASN_2_ hydrogels decreased considerably, and then remained almost unchanged; this indicates the formation of recoverable and stable networks during the latter part of the cyclic process.^[^ ^34^ ^]^ During the first cycle of the test, strong interfacial chemical bonds between the TSASNs and polymer chains were broken; subsequently, the reversible hydrogen bonds were regenerated, and the dissipated energy remained almost unchanged.^[^ ^35^ ^]^ The maximum tensile stress remained almost unchanged after 10 cycles (Figure 3g), indicating stable mechanical properties with excellent self‐recovery. The compression recovery of the PAM‐TSASN_2_ hydrogels was investigated by consecutive load‐unload compression experiments at 50% strain (Figure 3h). In the cyclic compression tests, all the curves overlapped, except for the curve of the first cyclic process (which was a little larger than the remaining curves). The dissipated energy and maximum stress of the PAM‐TSASN_2_ hydrogels did not decrease significantly on testing (Figure 3i), indicating outstanding elastic recovery and stability. These results could be attributed to the rapid rupture and recombination of a large number of weak hydrogen bonds in the PAM‐TSASN_2_ hydrogels.^[^ ^33^ ^]^ To investigate the durability and stability of the PAM‐TSASN_2_ hydrogels further, a constant strain of 200% was applied for 100 consecutive load‐unload tensile cycles (Figure S5a, Supporting Information). Under these conditions, all the curves of the load–unload cyclic experiments almost completely overlapped, and the maximum tensile force remained almost constant for 100 cycles (Figure S5b, Supporting Information), indicating excellent fatigue resistance and reliability during long‐term application. Notably, the elastic recovery for 100 cycles was more than 90% (Figure S5c, Supporting Information). Subsequently, 100 consecutive load‐unload compression experiments were carried out under 50% strain (Figure S5d, Supporting Information). Under these conditions, the PAM‐TSASN_2_ hydrogels showed almost stable compressive strength and high elastic recovery (more than 85%) (Figure S5e,f, Supporting Information), indicating excellent elasticity and fatigue resistance. In general, the hysteresis loop of the PAM‐TSASN_2_ hydrogels remained almost constant during repetitive tensile and compressive cycling, indicating excellent durability. Thus, during repetitive cycling, the TSASNs homogenously diffused the external force, acting as stress‐transfer centers, while the rapid fracture and recombination of hydrogen bonds maintained the integrity and robustness of the three‐dimensional network structure of the PAM‐TSASN_2_ hydrogels.

As expected, the PAM‐TSASN_2_ hydrogels showed high flexibility, stretch‐ability, and compressibility. Moreover, they could withstand large stretching, twisting stretching, and knotting stretching (Figure S6a, Supporting Information), indicating robust mechanical properties. The PAM‐TSASN_2_ hydrogels could withstand biaxial stretching and more than 500% surface strain without breaking (5 × 5 cm^2^, Figure S6b, Supporting Information). Additionally, the hydrogels exhibited strong puncture resistance. The PAM‐TSASN_2_ hydrogel sheets withstood fierce puncturing by a sharp scalpel blade with no observable damage, and cylindrical PAM‐TSASN_2_ hydrogels, which were violently cut from top to bottom, gradually regained their original state (Figure S6c, Supporting Information), indicating efficient energy dissipation. Notably, PAM‐TSASN_2_ hydrogel strips (12 × 5 × 1.5 mm^3^) could lift 500 g of weight without breaking (Figure S6d, Supporting Information), indicating excellent mechanical properties and an outstanding ability to bear weight, which are essential properties of flexible electronic sensors.

### 2.3 Mechanical Properties of PAM‐TSASN_2_‐LiCl*_y_* Hydrogels

**Figure 4:**
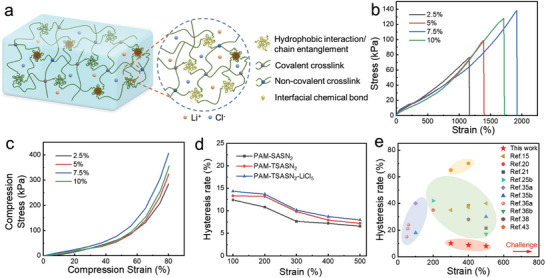
a) Schematic diagram of the interactions that occur in PAM‐TSASN_2_‐LiCl*_y_* hydrogels. b) Tensile and c) compressive stress‐strain curves of the PAM‐TSASN_2_‐LiCl*_y_* hydrogels. d) Hysteresis rates of the PAM‐SASN_2_, PAM‐TSASN_2_, and PAM‐TSASN_2_‐LiCl_5_ hydrogels at different strains. e) Comparison of the hysteresis performance of the PAM‐TSASN_2_‐LiCl_5_ hydrogels with those of previously reported hydrogels.

The PAM‐TSASN_2_ hydrogels, with excellent stretchability, elasticity, and fatigue resistance, exhibit high potential for the fabrication of wearable electronic devices. Subsequently, PAM‐TSASN_2_‐LiCl*_y_* hydrogels were fabricated by adding LiCl (to provide conductive ions) into the PAM‐TSASN_2_ hydrogels (**Figure** **4**a). XPS analysis confirmed the presence of Li and Cl in the PAM‐TSASN_2_‐LiCl*_y_* hydrogels, confirming their fabrication by the incorporation of LiCl into PAM‐TSASN_2_ hydrogels (Figure S7, Supporting Information). Additionally, as shown in Figure 4a, the PAM‐TSASN_2_‐LiCl*_y_* hydrogels contained hydrogen bonds, hydrophobic interactions, polymer chain entanglement, and strong interfacial chemical bonds, which increased their tensile and compressive strength.^[^ ^37^ ^]^ As shown in Figure 4b, on increasing the mass fraction of LiCl from 2.5% to 5%, and then to 7.5%, the fracture strength of the PAM‐TSASN_2_‐LiCl*_y_* hydrogels increased from 80 to 100 kPa, and finally to 130 kPa. The same trend was observed for the compression curve (Figure 4c). Moreover, the addition of LiCl improved the fracture strain of the PAM‐TSASN_2_ hydrogels, possibly because LiCl cleaves partial hydrogen bonds, causing polymer‐chain entanglement.^[^ ^30^ ^]^ On increasing the mass fraction of LiCl to 10%, the break stress and compressive stress of the hydrogels decreased (Figure 4b,c), possibly due to precipitation caused by a high concentration of LiCl, causing hydrogel heterogeneity.^[^ ^38^ ^]^ Continuous load‐unload cycles of the PAM‐TSASN_2_‐LiCl_5_ hydrogels (Figure S8a,b, Supporting Information) at different strains indicated that the dissipated energy during each cycle was slightly more than that of the PAM‐TSASN_2_ hydrogels, possibly due to the strong hydration of Li^+^.^[^ ^39^ ^]^ Similarly, the hysteresis of the PAM‐TSASN_2_‐LiCl_5_ hydrogels was slightly higher than that of the PAM‐SASN_2_ hydrogels (Figure 4d), possibly due to the strong hydration of Li^+^. Interestingly, the hysteresis of the PAM‐TSASN_2_‐LiCl_5_ hydrogels decreased on increasing the strain, indicating excellent elasticity and ultra‐low hysteresis under high tensile conditions. Notably, the PAM‐TSASN_2_‐LiCl_5_ hydrogel sensors synthesized in this study show very low hysteresis compared to previously reported hydrogels (Figure 4e). As the gauge factor (GF) of the PAM‐TSASN_2_‐LiCl_5_ hydrogels increased up to 3.84 within a strain range of 500–800% (discussed in a later section), their durability and stability were analyzed under a constant strain of 200% for 100 consecutive load‐unload tensile cycles (Figure S9a, Supporting Information). The dissipated energy of the PAM‐TSASN_2_‐LiCl_5_ hydrogels decreased significantly after the first cycle, and then remained almost unchanged, indicating the formation of recoverable and stable networks during the latter part of the cyclic process. The maximum tensile stress and force remained almost unchanged for 100 cycles (Figure S9b,c, Supporting Information), indicating excellent mechanical properties, even after tensile cycling. Thus, the PAM‐TSASN_2_‐LiCl_5_ hydrogels exhibit high potential as flexible electronic devices.

### 2.4 Adhesion Properties

**Figure 5:**
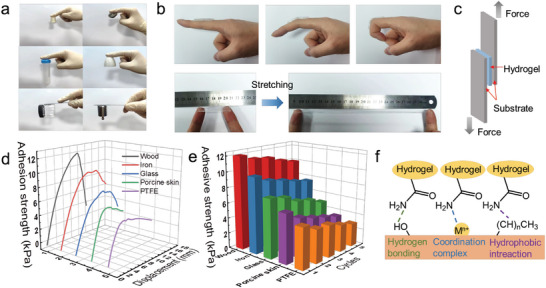
Adhesion properties of the PAM‐TSASN_2_‐LiCl_5_ hydrogels. a) Adhesion to different material surfaces. b) PAM‐TSASN_2_‐LiCl_5_ hydrogels directly adhere to the finger during bending and stretching. c) Schematic diagram for the lap‐shear adhesion test. d) Adhesion strength curves of the hydrogels to different substrates. e) Repeatable adhesion tests with different substrates. f) Schematic adhesion mechanism of the PAM‐TSASN_2_‐LiCl_5_ hydrogels.

Self‐adhesion is an important characteristic of flexible electronic sensors, because an excellent self‐adhesion ability facilitates the acquisition of accurate and steady signals. The PAM‐TSASN_2_‐LiCl_5_ hydrogels adhered to different substrates, such as rubber, stone, plastic, ceramic, glass, and steel, as shown in **Figure** **5**a, indicating good adhesive behavior. The strong adhesiveness of the PAM‐TSASN_2_‐LiCl_5_ hydrogels with the human skin facilitates an effective monitoring of joint movements. The PAM‐TSASN_2_‐LiCl_5_ hydrogels showed strong tissue adhesiveness; they directly adhered to the skin and underwent bending or stretching with the finger joint (Figure 5b). Moreover, the PAM‐TSASN_2_‐LiCl_5_ hydrogels tightly adhered to the back of the hand without any additional adhesive, and could be peeled from the skin without any residue (Figure S10, Supporting Information). The adhesive strength of the PAM‐TSASN_2_‐LiCl_5_ hydrogels was investigated by a lap‐shear tensile experiment (Figure 5c). As shown in Figure 5d, the maximum adhesive strength of the PAM‐TSASN_2_‐LiCl_5_ hydrogels with wood, steel, glass, porcine skin, and polytetrafluoroethylene (PTFE) was 12, 9, 6, 4, and 4 kPa, respectively. The roughness and porosity of the wood surface enabled hydrogels and TSASNs to penetrate through it, resulting in strong adhesion (12 kPa).^[^ ^31^ ^]^ This adhesion was reliable and stable, possibly due to reversible physical interactions between the PAM‐TSASN_2_‐LiCl_5_ hydrogels and substrates.^[^ ^40^ ^]^ After five cycles, the adhesion strength reduced slightly, owing to some hydrogel‐surface damage after each cycle (Figure 5e). The steady and strong adhesion of the PAM‐TSASN_2_‐LiCl_5_ hydrogels with different substrates can be attributed to multiple covalent/non‐covalent bond interactions of the hydrogels, such as hydrogen bonding, coordination‐bond formation, and hydrophobic interactions (Figure 5f).^[^ ^41^ ^]^ Additionally, the PAM‐TSASN_2_‐LiCl_5_ hydrogels were highly transparent, with a transmittance of 88%, facilitating the observation of skin conditions on direct attachment to the skin (Figure S11, Supporting Information). Hence, the PAM‐TSASN_2_‐LiCl_5_ hydrogels could detect accurate motion signals as flexible electronic sensors.

### 2.5 Sensing Performance of PAM‐TSASN_2_‐LiCl_5_ Hydrogels

**Figure 6:**
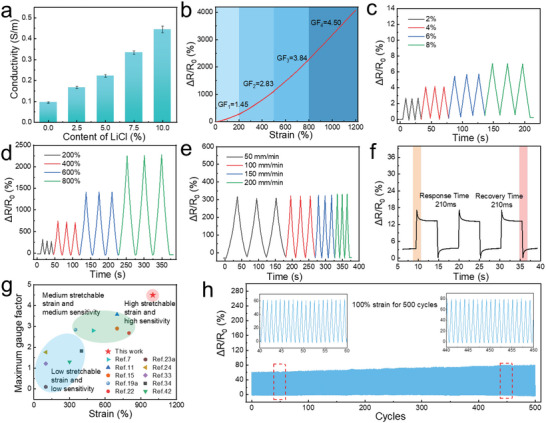
a) Conductivity of the PAM‐TSASN_2_‐LiCl*_y_* hydrogels (*n* = 3, *n* is the sample size for each group). b) Relative resistance changes and gauge factor of the PAM‐TSASN_2_‐LiCl_5_ hydrogels at different strains. c,d) Relative resistance changes of the PAM‐TSASN_2_‐LiCl_5_ hydrogels at small (2–8%) and large (200–800%) strains. e) Relative resistance changes of the PAM‐TSASN_2_‐LiCl_5_ hydrogels when stretched to 200% at different strain rates. f) Response and recovery times of the PAM‐TSASN_2_‐LiCl_5_ hydrogels. g) Comparison of the maximum gauge factor of the PAM‐TSASN_2_‐LiCl_5_ hydrogels with those of previously reported hydrogels. h) Relative resistance changes of the PAM‐TSASN_2_‐LiCl_5_ hydrogels for 500 cycles at a strains of 100%.

Ideal hydrogel sensors exhibit outstanding stretchability, high strain sensitivity, ultrafast response, and excellent electrical stability. The electrical behavior of the PAM‐TSASN_2_‐LiCl*_y_* hydrogels were systematically investigated, to explore their potential utility as wearable sensors. LiCl addition enhanced the mechanical strength of the PAM‐TSASN_2_‐LiCl_5_ hydrogels, and improved their conductivity and electrical sensitivity. Under an electric field, the Li^+^ and Cl^−^ inside the hydrogels migrated in opposite directions (to the cathode and anode), generating currents.^[^ ^43^ ^]^ As shown in **Figure** **6**a, the ionic conductivity of the PAM‐TSASN_2_‐LiCl*_y_* hydrogels increased from 0.1 to 0.4 S m^−1^ on increasing their LiCl content, possibly due to enhanced ion movement in the hydrogel network and the formation of numerous ion‐migration channels.^[^ ^44^ ^]^ Tuning the LiCl content also affected the maximum gauge factor (GF). The GF indicates the sensitivity of hydrogel sensors; it is evaluated from the slope of the relative resistance changes (Δ*R*/*R*_0_) versus the strain curve.^[^ ^45^ ^]^ Here, at an LiCl content of 5%, the maximum GF was 4.5 (Figures S12 and S13, Supporting Information). The hydrogel sensors showed four diverse GFs (1.45, 2.83, 3.84, and 4.50) in the strain ranges of 0–200%, 200–500%, 500–800%, and 800–1200%, respectively, as shown in Figure 6b. Consequently, the PAM‐TSASN_2_‐LiCl_5_ hydrogels were investigated further; the hydrogel sensors showed high sensitivity in the small and large strain zones, indicating high applicability for the reliable monitoring of human activities.^[^ ^46^ ^]^ For a visual verification of sensitivity, an LED bulb was integrated into a circuit with the PAM‐TSASN_2_‐LiCl_5_ hydrogels (Figure S14, Supporting Information). On increasing the tensile strain from 0% to 1200%, the luminance of the LED bulb gradually diminished, indicating an increase in the resistance of the hydrogel sensors, possibly due to an elongation and narrowing of the Li^+^ and Cl^−^ transport pathways under tensile deformation.^[^ ^47^ ^]^ The repeatability of the hydrogel sensors was evaluated in the small‐strain (2–8%) (Figure 6c) and large‐strain (200–800%) regions (Figure 6d). On stretching the PAM‐TSASN_2_‐LiCl_5_ hydrogels thrice at each strain, similar Δ*R*/*R*_0_ curves were observed, indicating excellent stability and repeatability in the entire strain range. Thus, the hydrogel sensors could function under an extensive strain range.^[^ ^48^ ^]^ Frequency dependence is a significant feature of strain sensors.^[^ ^49^ ^]^ As shown in Figure 6e, no significant frequency‐related peak loss or hysteresis was observed at different tensile speeds (from 50 to 200 mm min^−1^), indicating high signal stability. Additionally, the response and recovery times of the PAM‐TSASN_2_‐LiCl_5_ hydrogels were both 210 ms (Figure 6f), indicating negligible hysteresis and rapid response, which facilitates real‐time detection. A comparison of the maximum GF of the PAM‐TSASN_2_‐LiCl_5_ hydrogel sensor with those of previously reported hydrogel sensors indicates that the former exhibits a higher sensitivity, and is applicable over a wider strain‐range (Figure 6g). Notably, hydrogel sensors should exhibit high reliability and fatigue resistance for the effective long‐term monitoring of human activities. The PAM‐TSASN_2_‐LiCl_5_ hydrogels showed favorable repeatability with negligible fluctuation, and retained a good amplitude and waveform after 500 consecutive cycles at 100% strain (Figure 6h). During long‐term durability testing, despite a slight increase in the △*R*/*R*_0_ signals due to water evaporation, the hydrogels showed acceptable durability and reliability for long‐term practical application.

**Figure 7:**
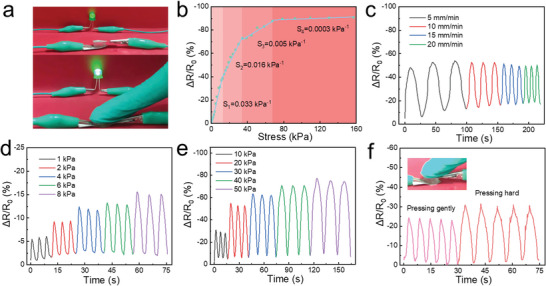
a) Change in the LED brightness, before and after a finger pressed the PAM‐TSASN_2_‐LiCl_5_ hydrogels. b) Relative resistance changes and pressure sensitivity at various pressures. c) Relative resistance changes of the PAM‐TSASN_2_‐LiCl_5_ hydrogels at a pressure of 20 kPa, with different compression speeds. d,e) Relative resistance changes of the PAM‐TSASN_2_‐LiCl_5_ hydrogels under low (1–8 kPa) and high (10–50 kPa) pressures. f) Relative resistance changes observed when a finger presses the PAM‐TSASN_2_‐LiCl_5_ hydrogels.

The PAM‐TSASN_2_‐LiCl_5_ hydrogels could also be used as pressure sensors. As shown in **Figure** **7**a, the LED brightness was significantly enhanced when a finger pressed the hydrogels, possibly due to an ion‐migration‐rate enhancement and migration‐path shortening because of the applied pressure. *S* (pressure sensitivity of the hydrogel sensors) was estimated from the slope of the relative resistance changes (Δ*R*/*R*_0_) versus pressure curve (Figure 7b). The hydrogel‐sensor sensitivity exhibited four different regions: 0.033 kPa^−1^ (within 0–12 kPa), 0.016 kPa^−1^ (within 12–35 kPa), 0.005 kPa^−1^ (within 35–70 kPa), and 0.0003 kPa^−1^ (within 70–150 kPa). Additionally, as shown in Figure 7c, the Δ*R*/*R*_0_ signals were independent of the compression speed, indicating excellent hydrogel‐sensor stability. Thus, the hydrogel sensors showed excellent stability and repeatability under low (1–8 kPa) and high (10–50 kPa) pressure (Figure 7d,e), indicating high applicability as pressure sensors to monitor human motion. The Δ*R*/*R*_0_ signals of the PAM‐TSASN_2_‐LiCl_5_ hydrogel sensors decreased on increasing the finger pressure (Figure 7f); moreover, the sensors showed good long‐term compression‐cycling stability (Figure S15, Supporting Information). Thus, the PAM‐TSASN_2_‐LiCl_5_ hydrogel sensors showed high sensitivity and good stability, indicating high potential as flexible wearable sensors.

### 2.6 Application of PAM‐TSASN_2_‐LiCl_5_ Hydrogels for Human Activity Monitoring

**Figure 8:**
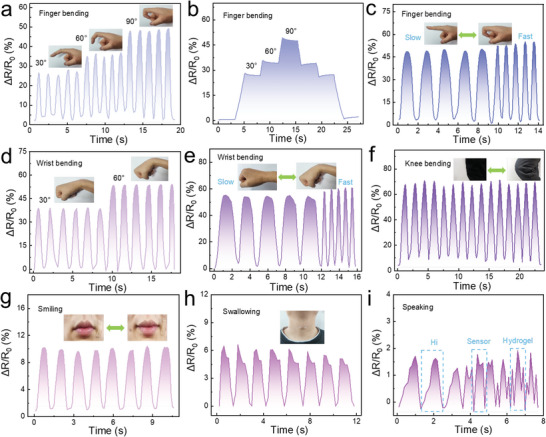
Real‐time relative resistance changes of the PAM‐TSASN_2_‐LiCl_5_ hydrogel sensors for monitoring the following large and subtle human movements: a,b) finger bending at different angles, c) finger bending at different speeds, d) wrist bending at different angles, e) wrist bending at different speeds, f) knee bending, g) smiling, h) swallowing, and i) saying “Hi,” “Sensor,” and “Hydrogel.”

The PAM‐TSASN_2_‐LiCl_5_ hydrogel sensors are useful to detect different human movements for health evaluation. The relative resistance changes (△*R*/*R*_0_) of the hydrogel sensors improved on increasing the finger‐bending angle from 30° to 90°; when the finger was straightened, the △*R*/*R*_0_ signals immediately recovered its original state, as shown in **Figure** **8**a. Thus, the PAM‐TSASN_2_‐LiCl_5_ hydrogel sensors showed high sensitivity and repeatability. The relative‐resistance signals remained almost unchanged when the finger was maintained at a constant angle of 30°, 60°, or 90° (Figure 8b), indicating that the PAM‐TSASN_2_‐LiCl_5_ hydrogel sensors showed high electrical stability. The speed of finger bending corresponded to the width of the electrical signal waveform (Figure 8c). Similarly, the PAM‐TSASN_2_‐LiCl_5_ hydrogel sensors could rapidly, precisely, and repeatedly examine wrist‐bending movements at different angles and speeds (Figure 8d,e). Although the △*R*/*R*_0_ signals changed slightly for wrist bending, because the wrist cannot be accurately bent to 30° or 60°, the hydrogel sensors could accurately monitor changes in the wrist angles. In Figure 8f, the △*R*/*R*_0_ signals of the PAM‐TSASN_2_‐LiCl_5_ hydrogel sensors also changed rapidly and precisely during knee‐joint movement; this can be used for the monitoring of joint diseases. In addition to large‐scale movements, small muscle motions (such as those that occur during smiling and swallowing; Figure 8g,h) could be accurately detected by repeatable electrical signals. Moreover, the PAM‐TSASN_2_‐LiCl_5_ hydrogel sensors were applied for phonation recognition. As shown in Figure 8i, the pronunciation of different words could be distinguished by the hydrogels, possibly by sensing the vibration of the vocal cord while uttering them. The sound of each word (“Hi,” “Sensor,” and “Hydrogel”) generated a specific and repeatable signal. These results confirm the high potential of the PAM‐TSASN_2_‐LiCl_5_ hydrogel sensors for practical application.

## 3 Conclusion

Summarizing, this study describes the synthesis of novel nanocomposite hydrogels with super elasticity and low hysteresis through the introduction of TSASNs into a PAM network. Due to the fractal structure of TSASNs and strong interfacial chemical bonding between the TSASNs and PAM, the PAM‐TSASN_2_ hydrogels showed excellent mechanical properties, including high stretch‐ability (with a breaking strain of 1200%), excellent mechanical properties (with a tensile stress of 80 kPa), outstanding compressive properties (they could withstand 80% strain without breaking), and high fatigue resistance. The introduction of LiCl into the PAM‐TSASN_2_ hydrogels improved their mechanical properties further (the modified hydrogels showed a fracture strength of 100 kPa), without affecting their low hysteresis and high stability. Moreover, LiCl enhanced their ionic conductivity (0.2 S m^−1^) and GF value (4.5). The conductive PAM‐TSASN_2_‐LiCl_5_ hydrogels showed excellent stability, with no electrical‐signal deterioration during 500 successive tensile cycles, along with efficient adhesion and good transparency (>90%), making them particularly suitable as wearable electrical sensors for the accurate sensing of different human activities. Therefore, this study describes the synthesis and outstanding properties of PAM‐TSASN_2_‐LiCl_5_ hydrogels that exhibit promising prospects for utilization as wearable electronics, in healthcare diagnosis, and as human–machine interfaces.

## 4 Experimental Section

### Materials

The monomer acrylamide, (AM, 99.0%, MW: 71.08 g mol^−1^), chemical cross‐linker, *N*,*N*′‐methylene dimethyl (acrylamide) (MBA, 97%, MW: 154.17 g mol^−1^), initiator, potassium persulfate (KPS, 99.99%, MW: 270.32 g mol^−1^), and lithium chloride (LiCl, ≥99%, MW: 42.39 g mol^−1^) were purchased from Shanghai Macklin Biochemical Co., while 3‐(trimethoxysilyl) propyl methacrylate (TMSPMA, 97%, MW: 248.35 g mol^−1^), the organic amine, tris (hydroxymethyl) ‐aminomethan (Tris, 99.9%, MW: 121.14 g mol^−1^), emulsifier (CTATos, 97%, MW: 248.35 g mol^−1^), ethanol (95%, MW: 46.07 g mol^−1^), and tetraethyl orthosilicate (TEOS, 98%, MW: 208.33 g mol^−1^) were purchased from Shanghai Aladdin Biochemical Technology Co. All the materials and reagents were used as received, without further purification.

### Preparation of TSASNs

To synthesize the TSASNs, 2.5 g of super arborized silica nanoparticles (SASNs) (the synthesis of SASNs is provided in Supporting Information) was added to 400 mL of an ethanol/deionized water mixture (3/1, V/V), and hydrolyzed by ultrasonic agitation for 1 h. The pH of the mixture was adjusted to 4.0 using oxalic acid. Subsequently, a specific amount of TMSPMA (0.1 times the weight of SASN) was added into this mixture, fully dispersed under high‐speed shear, and stirred at 70 °C for 4 h. After the reaction, a mixture of ethanol/water (1/1, V/V) was used for centrifugation cleaning (repeated thrice), to remove the TMSPMA physically adsorbed onto the SASN surface and reaction by‐products. The cleaned particles were dried in a vacuum drying oven at 60 °C for 6 h to obtain the TMSPMA‐grafted SASNs, labeled TSASNs.

### Preparation of PAM‐TSASN‐LiCl Hydrogels

LiCl was added to the PAM‐TSASN_2_ hydrogels (the fabrication of PAM‐TSASN*_x_* hydrogels has been described in Supporting Information), to produce hydrogels with high adhesion and conductivity. First, AM (2.4 g) and TSASN_2_ (48 mg) were dissolved in 15 mL of deionized water to produce a transparent solution. Subsequently, LiCl*_y_* (*y* = 2.5, 5, 7.5, and 10 wt% of AM), 100 µL of an MBA solution (10 mg mL^−1^), and 150 µL of a KPS solution (50 mg mL^−1^) were mixed into it and homogenized (by magnetic agitation and ultrasound). After degassing with N_2_, the final solution was injected into a homemade quartz mold and the polymerized at 60 °C for 1 h, generating the PAM‐TSASN_2_‐LiCl*_y_* hydrogel. The composition of the hydrogel is summarized in Table S2, Supporting Information.

### Informed Consent

The experimental description of human subjects (Figures 5 and 8; Figure S10, Supporting Information) was provided after obtaining informed consent from the volunteers. It was confirmed that the use of wearable devices for research does not require the approval of an institutional review board.

### Statistical Analysis

All quantitative data are expressed as the mean ± standard deviation of at least three independent parallel experiments.

The detailed characterization, mechanical measurements, self‐adhesion measurements, and electrical measurements have been placed in the Supporting Information.

## Conflict of Interest

The authors declare no conflict of interest.

## Supporting information

Supporting Information

## Data Availability

The data that support the findings of this study are available from the corresponding author upon reasonable request.
