# Supplementary material for: Surface Modification of Super Arborized Silica for Flexible and Wearable Ultrafast‐Response Strain Sensors with Low Hysteresis
Source: Adv Sci (Weinh). 2023 Jun 28;10(25):2301713. doi: 10.1002/advs.202301713 (PMC10477872; doi:10.1002/advs.202301713)
Supplement: Supplementary file 1 — Supporting Information [file ADVS-10-2301713-s001.pdf]

ADVANCED SCIENCE
Open Access

Supporting Information

for *Adv. Sci.*, DOI 10.1002/advs.202301713

Surface Modification of Super Arborized Silica for Flexible and Wearable Ultrafast-Response Strain Sensors with Low Hysteresis

Shaowei Han, Huanhuan Tan, Jia Wei, Hang Yuan, Songwei Li, Peipei Yang*, Haoyang Mi, Chuntai Liu and Changyu Shen*

Supporting Information

Surface Modification of Super Arborized Silica for Flexible and Wearable Ultrafast-Response Strain Sensors with Low Hysteresis

Shaowei Han[a], Huanhuan Tan[a], Jia Wei[b], Hang Yuan[a], Songwei Li[a], Peipei Yang[a*], Haoyang Mi[a], Chuntai Liu[a], Changyu Shen[a]*

[a] National Engineering Research Center for Advanced Polymer Processing Technology, Key Laboratory of Materials Processing and Mold (Ministry of Education), Zhengzhou University, Zhengzhou 450002, China.

[b] Yunnan Tobacco Quality Inspection & Supervision Station, Kunming 650106, China.

Corresponding authors: lisw@zzu.edu.cn; peipeiyang@zzu.edu.cn.

Experimental Section

Preparation of Super Arborized Silica Nanoparticle (SASN): Monodispersed SASNs were prepared by a modified method developed by Zhang K. et. al.[1] In a typical experiment, 4.8 g emulsifier CTATos and 0.71 g organic amine Tris were mixed with 250 mL water in a 500 mL beaker. The mixture was stirred at 80°C for 1 h to make sure all emulsifier and organic amine were dissolved completely. Then, 12.5 g TEOS was added into the flask quickly. After 2 h stirring with a speed of 1000 rpm at 80°C, the final slurry was poured into 150 mL ethanol. The SASNs were isolated by 3 min centrifugation with a speed of 3000 rpm. After that, the SASNs were re-dispersed into ethanol/water (1/1, V/V) mixture and then were isolated by

centrifugation (3000 rpm, 3 min) to remove the free emulsifier and organic amine. Then three cycles of re-dispersion in water/centrifugation (3000 rpm, 3min) were implemented to replace the ethanol in the dispersion for the next lyophilization procedure. The white powders obtained after the lyophilization were then transferred into furnace to remove the soft templates completely by 250 min calcination at 600℃. Finally, the pure SASNs were redispersed in water by intense ultrasonic treatment. Then, a surface activation process was performed on the SASNs by HF etching. SASN dispersion was mixed with dilute HF aqueous solution following molar ratio 100/0.5 (SASN / HF). After 2h stirring at ambient temperature, the mixture was transferred into dialysis tube with retention molecular weight of 10000 Da. The dialysis was finished when the pH of surrounding aqueous solution was 7.0. Then the SASN was dispersed into powder by rotary evaporation.

Preparation of PAM-TSASN Hydrogels: Firstly, monomer acrylamide (2.4 g) and TSASN were dissolved in 15 mL deionized water to obtain a transparent solution. And then, 100 µL MBA solution (10 mg/mL) and 150 µL KPS solution (50 mg/mL) were added and stirred to prepare homogenous solution. Finally, the resultant solution by degassing with N_2 was injected into a mold consisting of a pair of parallel glass plates and a silicone spacer (100 × 100 × 2 mm^3), and the polymerization took place at 60℃ for 1 h. The resulting hydrogel was defined as PAM-$TSASN_x$ hydrogel. For comparison, the PAM hydrogels and PAM-$SASN_x$ (x=1, 2, 3, and 4 wt % for AM) hydrogels were fabricated according to the above process. The composition of the hydrogels was provided in Table S1. The as-prepared samples were stored in a refrigerator at 10℃ for avoiding water volatilization.

Characterization: The chemical functional groups of the prepared monomers and polymers were characterized by FT-IR infrared spectrometer (Nicolet Nexus 870 spectrometer). After being dried to a constant weight, the different samples were mixed with KBr for the test with a 64-time scan, and the scan range between 4000 and 400 cm^{-1}. The chemical functional groups of the prepared monomers and polymers were characterized by X-ray photoelectron spectra

(Thermo Scientific K-Alpha). The transparency of PAM-$TSASN_2$-$LiCl_5$ hydrogels was tested from 800 to 400 nm by the UV-vis-NIR spectrophotometer (Lambda 1050+). The internal morphology of the hydrogel was investigated by using a scanning electron microscope (SEM instrument, ZEISS Gemini 300). The prepared hydrogel was frozen at -30℃ for 24 h, and then lyophilized at -60℃ for 72 h. The dried samples were soaked in liquid nitrogen and fractured to obtain an internal cross-section.

Mechanical measurement: The mechanical performances of the hydrogels were evaluated by UTM2203 electromechanical tester (Suns Technology Stock Co., Ltd.) at room temperature. The rectangular (12 × 4 × 1.5 mm^3) hydrogel sample was prepared, and each sample coated with silicone oil was directly tested for at least five times. The uniaxial tensile tests were carried out with the stretch rate of 50 mm/min with a 100 N load cell. For cyclic stretch test, silicone oil was applied to the surface of the hydrogel to prevent evaporation. The tensile strain was set at 100 % for 200 cycles and the stretch rate was set at 50 mm/min. The energy dissipation can be obtained by calculating the area of the hysteresis loop. Cylindrical hydrogel samples with a diameter of 10 mm and a height of 5 mm were used for compression tests. The test was carried out with a 100 N load cell (compression rate: 10 mm/min). For cyclic compression test, silicone oil was applied to the surface of the hydrogel to prevent evaporation. The compression strain was set at 50 % and the compression rate was set at 10 mm/min.

Adhesion measurement: Lap shear tensile tests were performed to evaluate the adhesion of the PAM-TSASN-LiCl hydrogels with different substrates.[2] Briefly, a rectangular hydrogel (20 mm × 20 mm) is attached between the surfaces of two substrates. The sample was then pulled at a fixed speed of 5 mm/min until it separated under ambient conditions. The adhesive strength was determined by dividing the maximum load by the corresponding overlap area. At least five specimens were tested for each hydrogel sample.

Electrical measurement: The electrical conductivities (σ) of hydrogels were measured using a digital multimeter (DMM4050, Tektronix). The values were calculated using the following equation:

$$\sigma = \frac{L}{RS}$$

where L is the gauge length, S refers to the cross-sectional area of the hydrogel sample and R is the resistance. The sensing properties were investigated by the combination of (UTM2203, Suns Technology Stock Co., Ltd.) and multimeter (DMM4050, Tektronix). As hydrogel sensors were stretched or compressed, their changes of real-time resistance were recorded by the above apparatuses. The relative changes of resistance were calculated by the formula:

$$\frac{\Delta R}{R_0} = \frac{R - R_0}{R_0}$$

where R_0 and R are the initial resistance and the resistance with applied strain, respectively. The gauge factor (GF) was defined as:

$$\mathrm{GF} = \frac{\frac{\Delta R}{R_0}}{\varepsilon}$$

$\frac{\Delta R}{R_0}$ is the relative change of resistance and ε is the applied strain.

The pressure sensitivity (S) was defined as:

$$\mathrm{S} = \frac{\frac{\Delta R}{R_0}}{\frac{\Delta P}{P_0}}$$

$\frac{\Delta R}{R_0}$ is the relative change of resistance and $\frac{\Delta P}{P_0}$ is the relative change of pressure.

In order to measure signals related to human activities, the PAM-TSASN-LiCl hydrogel strain sensors were attached on the relevant human epidermis to realize the synchronous detection.

Table S1. The compositions of the PAM-SASN hydrogels

Sample	AM (g)	SASN (mg)	MBA (10 mg/mL)	KPS (50 mg/mL)	Distilled H_2O (mL)
PAM	2.4	/	0.1 mL	0.15 mL	15
PAM-$SASN_1$	2.4	24	0.1 mL	0.15 mL	15
PAM-$SASN_2$	2.4	48	0.1 mL	0.15 mL	15
PAM-$SASN_3$	2.4	72	0.1 mL	0.15 mL	15
PAM-$SASN_4$	2.4	96	0.1 mL	0.15 mL	15

Table S2. The compositions of the PAM-TSASN-LiCl hydrogels

Sample	AM (g)	TSASN (mg)	LiCl (mg)	MBA (10mg/mL)	KPS (50mg/mL)	Distilled H_2O (mL)
PAM-$TSASN_2$	2.4	48	/	0.1 mL	0.15 mL	15
PAM-$TSASN_2$-$LiCl_{2.5}$	2.4	48	60	0.1 mL	0.15 mL	15
PAM-$TSASN_2$-$LiCl_5$	2.4	48	120	0.1 mL	0.15 mL	15
PAM-$TSASN_2$-$LiCl_{7.5}$	2.4	48	240	0.1 mL	0.15 mL	15
PAM-$TSASN_2$-$LiCl_{10}$	2.4	48	480	0.1 mL	0.15 mL	15

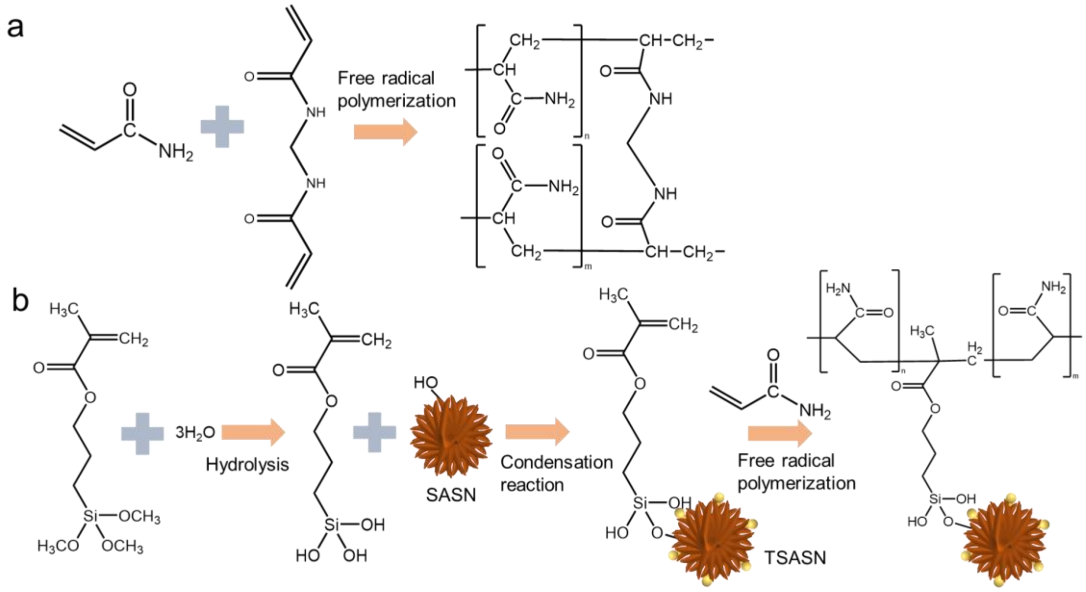

Figure S1. (a) Schematic illustration of the chemical reaction process of the AM and MBA. (b)The hydrolysis reaction of the TMSPMA, the grafting reaction process of SASN, and the chemical reaction process of the PAM-TSASN$_2$ hydrogels.

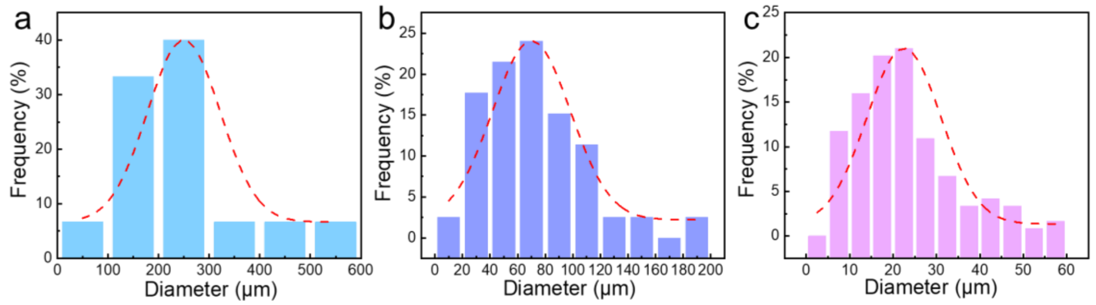

Figure S2. Diameter distribution histograms as well as Gaussian fitting curves of freeze-dried (a) PAM, (b) PAM-TSASN$_2$, and (c) PAM-TSASN$_2$-LiCl$_5$ hydrogels.

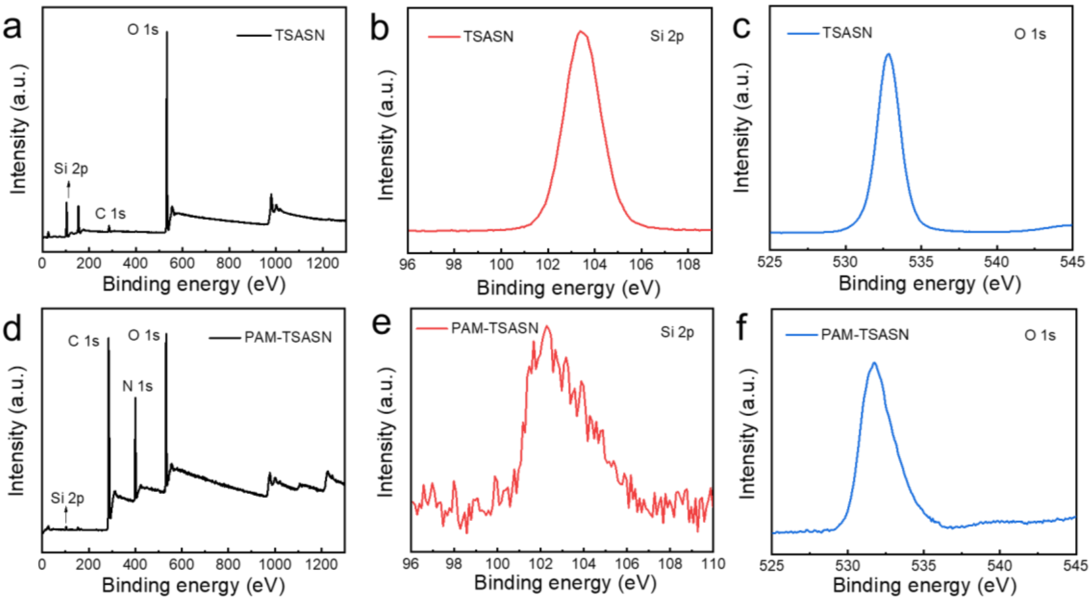

Figure S3. (a) XPS spectrum of TSASN. (b) High-resolution XPS spectra of the TSASN for Si 2p, and (c) O 1s. (d) XPS survey spectrum of the PAM-TSASN$_2$ hydrogels. (e) High-resolution XPS spectra of the PAM-TSASN$_2$ hydrogels for Si 2p, and (f) O 1s.

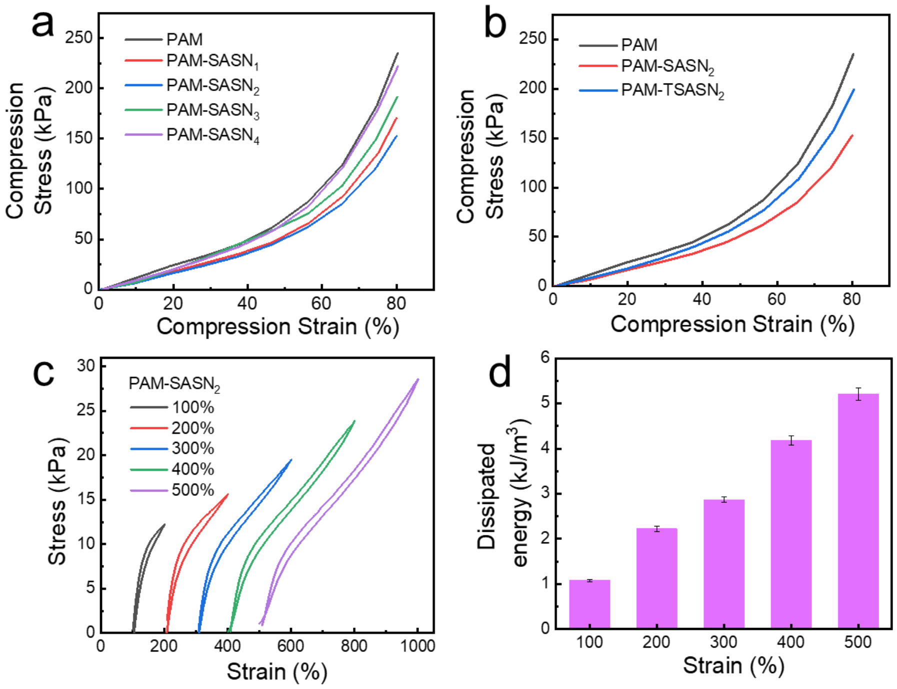

Figure S4. (a) Compression stress-strain curves of the PAM-SASN$_x$ hydrogels with different SASN contents. (b) Compression stress-strain curves of the PAM-SASN$_2$ hydrogels and PAM-

TSASN$_2$ hydrogels. (c) Load-unload tests at different strains of PAM-SASN$_2$ hydrogels, and (d) the corresponding dissipated energy of PAM-SASN$_2$ hydrogels (n = 3, n is the sample size for each group).

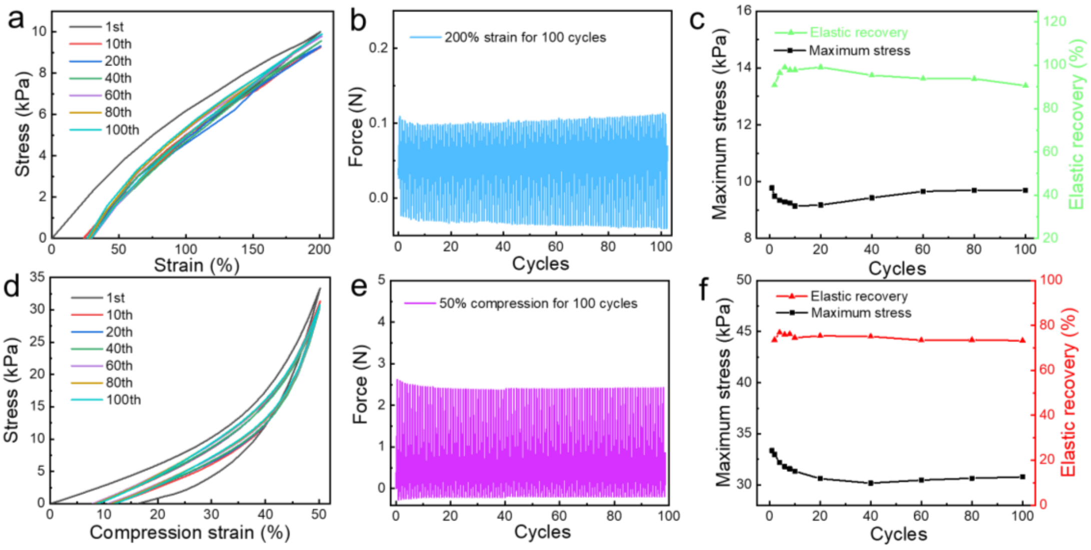

Figure S5. The mechanical performance of the PAM-TSASN$_2$ hydrogels: (a) successive tensile tests (100 cycles) with the strain of 200 %. (b) load cycle curve of 100 successive tensile cycles. (c) maximum stress and elastic recovery versus tensile cycles. (d) successive compression tests (100 cycles) with the strain of 50 %. (e) load cycle curve of 100 successive compression cycles. (f) maximum stress and elastic recovery versus compression cycles.

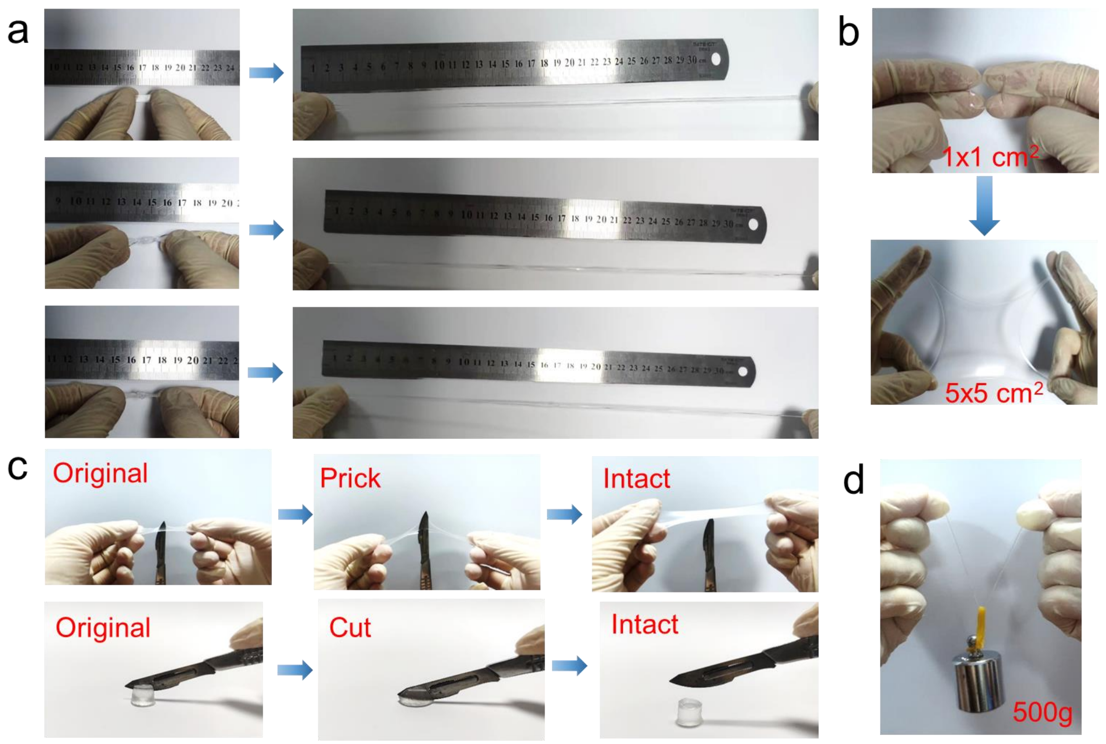

Figure S6. Photographs of the PAM-TSASN$_2$ hydrogels exhibiting excellent mechanical properties: (a) stretching, twisting stretching and knotting stretching. (b) Biaxial tension of a square PAM-TSASN$_2$ hydrogels. (c) Photographs of cutting PAM-TSASN$_2$ hydrogels from top to bottom with a sharp knife and puncturing the hydrogels via a sharp knife. (d) Photographs showing that the PAM-TSASN$_2$ hydrogels could lift a weight of 500 g.

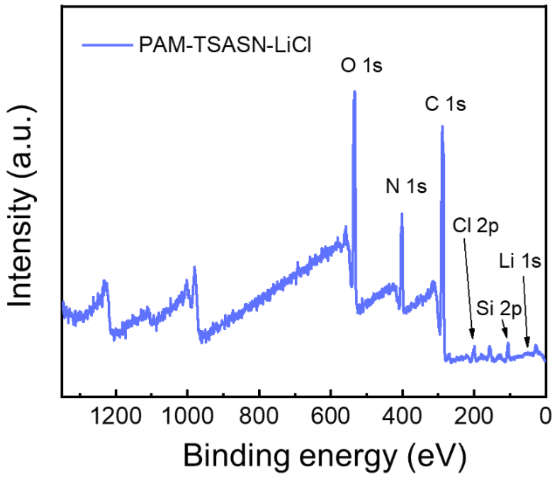

Figure S7. XPS spectra of the PAM-TSASN$_2$-LiCl$_5$ hydrogels.

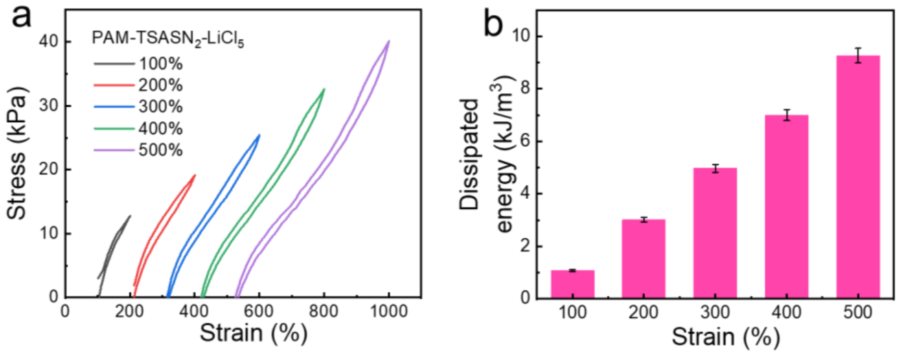

Figure S8. (a) Load-unload tests at different strains of PAM-TSASN$_2$-LiCl$_5$ hydrogels, and (b) the corresponding dissipated energy (n = 3, n is the sample size for each group).

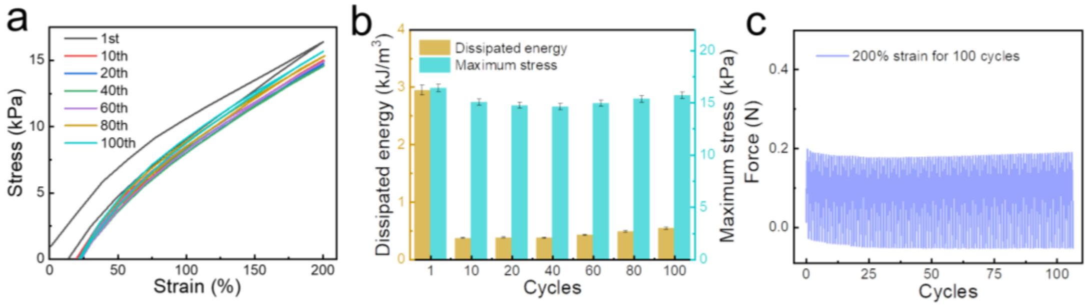

Figure S9. (a) The cyclic tensile curves of PAM-TSASN$_2$-LiCl$_5$ hydrogels under the tension of 200 % over 100 cycles, and (b) the corresponding maximum stress and dissipated energy (n = 3, n is the sample size for each group). (c) load cycle curve of 100 successive tensile cycles.

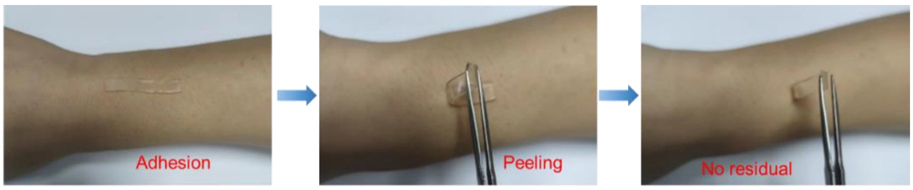

Figure S10. Exhibition of no residual when the PAM-TSASN$_2$-LiCl$_5$ hydrogels removed from arm.

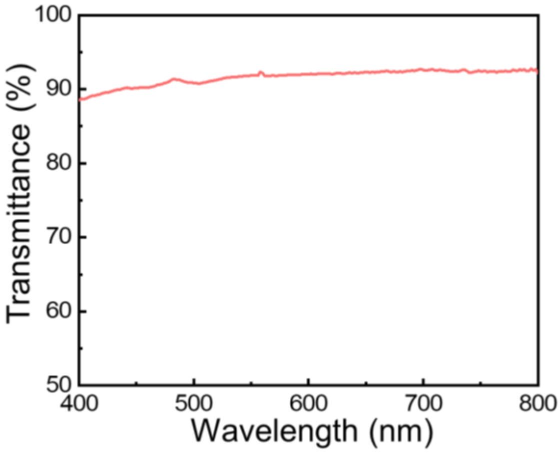

Figure S11. The transparency of PAM-TSASN$_2$-LiCl$_5$ hydrogels.

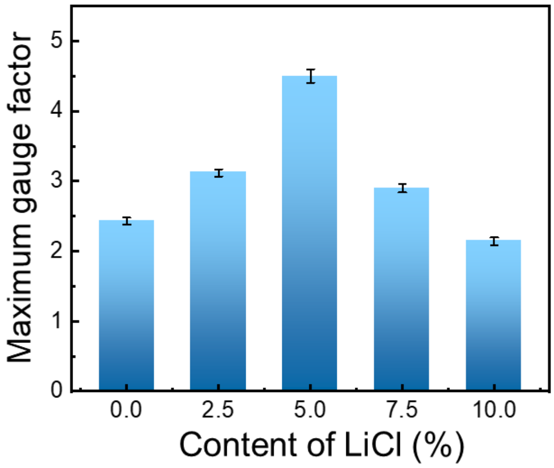

Figure S12. Maximum gauge factor of PAM-TSASN$_2$-LiCl$_y$ hydrogels (n = 3, n is the sample size for each group).

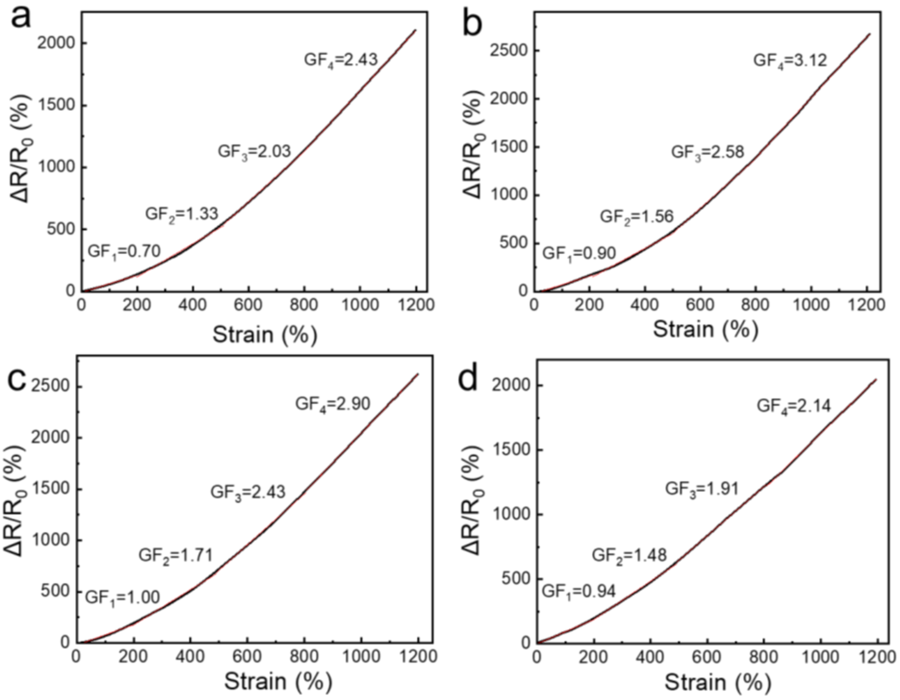

Figure S13. Relative resistance changes and gauge factor of the PAM-TSASN$_2$-LiCl$_y$ (y=0 %,2.5 %,7.5 %, and 10 %) hydrogels at different strains.

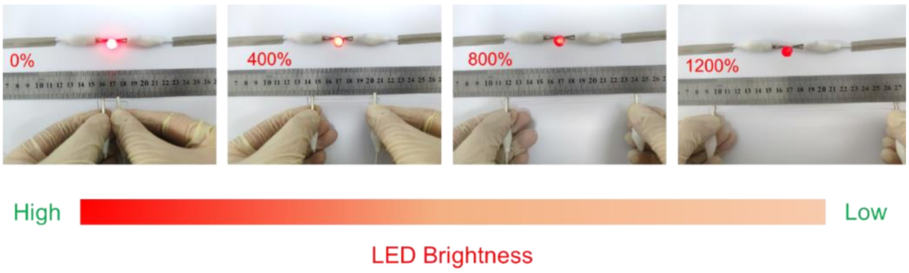

Figure S14. Change in LED brightness with the elongation of the PAM-TSASN$_2$-LiCl$_5$ hydrogels connected in the electric circuit.

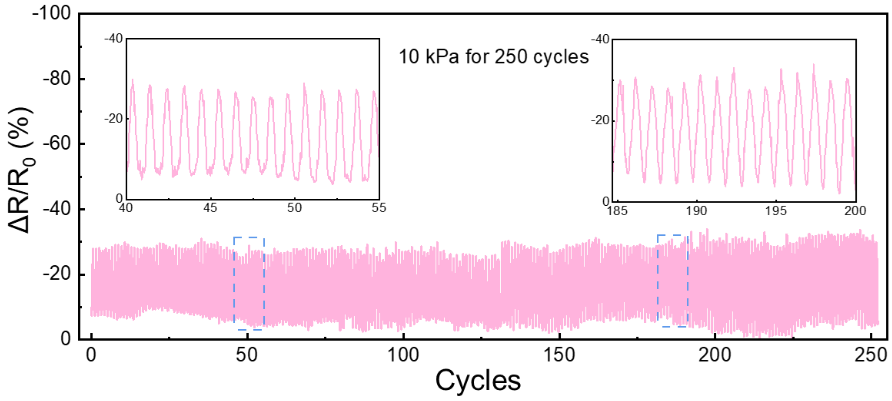

Figure S15. Relative resistance changes of the PAM-TSASN$_2$-LiCl$_5$ hydrogels for 250 cyclic compression curves at 10 kPa.

References

[1] K. Zhang, L. L. Xu, J. G. Jiang, N. Calin, K. F. Lam, S. J. Zhang, H. H. Wu, G. D. Wu, B. Albela, L. Bonneviot, P. Wu, *J. Am. Chem. Soc.* **2013**, *135* , 2427-2430.

[2] H. Zhang, H. Shen, J. Lan, H. Wu, L. Wang, J. Zhou, *Carbohydr. Polym.* **2022**, *295*, 119848.
